# Application of peripheral blood cytokine and immunoglobulin detection in ACTH therapy for the treatment of infantile spasms

**DOI:** 10.3389/fped.2024.1365917

**Published:** 2024-07-10

**Authors:** Xiaocui Wang, Qin Huang, Lulu Wu, Yang Yang, Xiaofei Ye, Bin Yang

**Affiliations:** Department of Neurology, Anhui Provincial Children’s Hospital, Hefei, Anhui, China

**Keywords:** infantile spasm, adrenocorticotropic hormone, immunoglobulin, cytokines, lymphocyte subsets

## Abstract

**Objective:**

This research aims to investigate the levels of lymphocytes, immunoglobulins, and cytokines in children with infantile spasms (IS) before and after adrenocorticotropic hormone (ACTH) therapy and to explore the application of these markers in evaluating the therapeutic effects of ACTH on infantile spasms.

**Methods:**

From May to November 2022, 35 children initially diagnosed with IS and treated at our hospital were regarded as the observation group, and 35 healthy children who underwent physical examination at our hospital during the same period were regarded as the control group. Children in the observation group received intramuscular injections of ACTH for 2 weeks. Fasting venous blood was collected from the control group and the observation group before and after ACTH therapy. Serum levels of immunoglobulins IgG, IgA, and IgM in serum were detected by immunoturbidimetry. T-cell subsets (CD3^+^, CD3^+^CD4^+^, and CD3^+^CD8^+^) and B-cell subsets [CD3^−^CD19^+^ and CD3^−^CD16^+^CD56^+^ natural killer (NK) cells] were detected by flow cytometry, and the ratio of CD3^+^CD4^+^/CD3^+^CD8^+^ was calculated. Serum levels of interleukin-1β (IL-1β), interleukin-2R (IL-2R), and interleukin-6 (IL-6) cytokines were detected by the enzyme-linked immunosorbent assay, and changes in serum cytokine and immunoglobulin levels in the two groups were compared before therapy, whereas in observation group one, these comparisons were made both before and after ACTH therapy.

**Results:**

Compared to the control group, the observation group showed significantly increased serum levels of immunoglobulins IgG and IgM before therapy, while the level of IgA was significantly decreased (*p *< 0.05). Also, the percentage of CD3^−^CD19^+^ B cells was significantly increased, while the percentages of CD3^+^ T cells and CD3^+^CD4^+^ T cells were significantly decreased (*p* < 0.05). The percentages of CD3^+^CD8^+^ T cells, CD3^−^CD16^+^CD56^+^ NK cells, and CD3^+^CD4^+^/CD3^+^CD8^+^ cells did not change significantly (*p* > 0.05); the levels of cytokines IL-1 β, IL-2R, and IL-6 were significantly increased (*p* < 0.05). Compared to levels before treatment, the serum level of immunoglobulin IgG in the observation group after ACTH therapy was significantly reduced (*p* < 0.05), while the IgA and IgM levels did not change significantly (*p* > 0.05). The percentages of CD3^+^ T cells and CD3^+^CD4^+^ T cells were significantly increased, while the percentages of CD3^−^CD16^+^CD56^+^ NK cells and CD3^−^CD19^+^ B cells were significantly decreased (*p *< 0.05); however, the percentages of CD3^+^CD8^+^ T cells and the CD3^+^CD4^+^/CD3^+^CD8^+^ ratio did not change significantly (*p* > 0.05). Furthermore, the levels of cytokines IL-1 β, IL-2R, and IL-6 were significantly reduced (*p* < 0.05).

**Conclusion:**

Children with IS exhibit immune dysfunction, and the changes in serological immune indices after ACTH treatment indicate that ACTH may control seizures in IS children by regulating and improving immune dysfunction. Therefore, the therapeutic effects of ACTH on IS can be evaluated by detecting the levels of cytokines and immunoglobulins.

## Introduction

Infantile spasms (IS) represent the most common epilepsy syndrome during infancy (1), usually occurring within the first year of age, with a peak period ranging from 4 to 7 months of age; it has an incidence of 0.2‰–0.5‰ (2). IS is believed to have a multifactorial pathogenesis, involving a complex interplay of genetic, developmental, and environmental factors. Thus, early diagnosis and timely treatment are helpful for a radical cure and prognosis of spasticity in children affected by IS (3). Recent studies suggest that neuroinflammation and immune dysregulation may play a significant role in the onset and progression of IS (PMID: 1).

The causes of IS can be broadly categorized into genetic, structural, metabolic, and unknown (cryptogenic) origins. Some of the common genetic causes include trisomy 21 (Down syndrome), tuberous sclerosis complex (TSC), and other chromosomal abnormalities. Structural causes often involve brain malformations, hypoxic-ischemic encephalopathy, and intracranial hemorrhage. Metabolic disorders, such as phenylketonuria and mitochondrial diseases, are also known to cause IS. In many cases, the cause remains unknown, leading to its classification as cryptogenic. The heterogeneity of IS etiologies suggests that underlying causes may significantly influence the baseline immunological levels and response to treatment. For example, children with Down syndrome often exhibit altered immune function, characterized by abnormalities in T- and B-cell populations. Similarly, tuberous sclerosis complex, a genetic disorder that leads to the growth of benign tumors in multiple organs, including the brain, is associated with immune dysregulation and an increased risk of infection.

Adrenocorticotropic hormone (ACTH), recognized as a first-line drug for IS (4), has high efficiency, but its mechanism of action has not been fully understood. It is known to modulate the immune response, potentially mitigating the neuroinflammatory processes implicated in IS (PMID: 27199888). As a result, finding biomarkers that can accurately evaluate the therapeutic effects is of great clinical importance for guiding the diagnosis and treatment of IS patients (5).

In the in-depth study of immunology, it has been found that immune factors may be related to the pathogenesis of IS, and an imbalance of immune regulation is involved in its development (6). Peripheral blood cytokine levels, lymphocyte numbers, and immunoglobulin levels are critical indicators for studying immunological mechanisms (PMID: 28260995). Changes in the levels of T lymphocytes (CD3^+^, CD4^+^, and CD8^+^), B lymphocytes (CD3^−^CD19^+^), immunoglobulins (IgG, IgM, and IgA), and cytokines (IL-1β, IL-2R, and IL-6) often indicate immune deficiencies in the body.

In this study, we aimed to evaluate the immune function of children with IS before and after ACTH therapy by measuring serum levels of immunoglobulins, lymphocytes, and cytokines and to explore the immunological mechanism of ACTH in the treatment of IS.

## Methods

### General data

From May to November 2022, 35 children with IS who were initially diagnosed and received treatment in our hospital were regarded as the observation group (IS group), and 35 healthy children who underwent physical examination in our hospital during the same period were regarded as the control group. The observation group comprised 19 boys and 16 girls, aged between 2 and 18 months, with a mean age of 8.13 ± 4.29 months. The control group included 21 boys and 14 girls, aged between 3 and 16 months, with a mean age of 6.58 ± 2.37 months. There were no significant differences in gender (*p* = 0.63, >0.05) or age (*p* = 0.54, >0.05) between the two groups, making them comparable.

### Inclusion and exclusion criteria

The inclusion criteria are as follows: (1) Physical examination meeting the diagnostic criteria of IS (7, 8), characterized by transient generalized muscle spasms with the trunk and legs bending at the onset and both arms extending outward and forward; (2) Electroencephalogram (EEG) examination meeting the diagnostic criteria of IS (8), showing special peak rhythm disturbance changes at the onset; (3) cerebrospinal fluid examination showing increased albumin, decreased protein, and increased blood–brain barrier permeability; (4) newly diagnosed children who have not yet received any treatment; (5) children with complete medical records; and (6) children and their parents who have signed the informed consent form.

The exclusion criteria are as follows: (1) children who had received glucocorticoids and other immune drugs before treatment; (2) children with a history of allergy or contraindication to adrenocorticotropic hormone; (3) relevant examinations before enrollment that showed infection, abnormal liver and kidney function, or stress; (4) children suffering from diseases affecting the immune system, such as immune deficiency diseases, blood system diseases, and connective tissue diseases; and (5) children participating in other studies at the same time.

### Methods

In the observation group, children received ACTH (produced by Shanghai Shangyao Biochemical First Pharmaceutical Co., Ltd., Shanghai, China, batch number 1709601) via intramuscular injections. The dosage was set at 1 unit per kilogram, administered twice daily continuously for 2 weeks (9).

### Observation indicators and efficacy evaluation criteria

The treatment effects of ACTH were evaluated using spasm frequency as the observation index (10). After treatment, outcomes were categorized as follows: cases with no spasm attack were classified as “significant effect”; cases with a spasm attack frequency of ≤50% compared to before treatment were classified as “remission”; and cases with a spasm frequency >50% compared to before treatment were classified as “invalid.” The total effective ratio was calculated as follows: total effective ratio = [(number of significantly effective instances + number of remission instances)/total number of instances] × 100%.

### Sample collection and detection of indicators

The observation group received blood sampling once before and after treatment, while the children in the control group received blood sampling only once. In the morning, 1 ml of venous blood was drawn into an Ethylenediaminetetraacetic acid (EDTA) anticoagulant tube, and 3 ml of venous blood was drawn into a separating gel coagulation-promoting tube. The venous blood samples in the EDTA anticoagulant tube were centrifuged at 3,000 r/min for 10 min, and then the serum (supernatant) was extracted. The percentages of CD3^+^ T cells, CD3^+^CD4^+^ T cells, CD3^+^CD8^+^ T cells, CD3^−^CD16^+^CD56^+^ natural killer (NK) cells, and CD3^−^CD19^+^ B cells were measured and calculated using a Biosciences FACS Calibur flow cytometer (BD Biosciences, Franklin Lakes, NJ, USA). The venous blood samples in the separating gel coagulation-promoting tube were left at room temperature for 0.5 h to naturally coagulate and then centrifuged at 3,000 r/min for 10 min to extract the serum (supernatant), which was divided into two parts; one part was used to determine the levels of IgA, Immunoglobulin G (IgG), and IgM using a Beckman AU480 automatic biochemical analyzer (Beckman, Brea, CA, USA), while the other part was used to determine the serum levels of IL-1β, IL-2R, and IL-6 by the enzyme-linked immunosorbent assay (ELISA) method.

### Statistical methods

SPSS22.0 software and Origin software were used for statistical processing and drawing pictures, respectively. Measurement data were expressed as mean ± standard deviation and analyzed by the *t*-test for comparisons between the two groups; count data were expressed as frequency [*n* (%)] and analyzed by the χ^2^ test for comparisons between groups. *p* < 0.05 indicates statistical significance.

## Results

### Demographic and clinical characteristics of the two groups

The IS group comprised 35 patients, including 20 boys and 15 girls, with the age of onset ranging from 6 days to 1 year and 10 months (average 4.98 ± 4.19 months). Eighteen patients had previously taken one to three antiepileptic drugs without successful seizure control. Genetic testing was conducted on 16 patients, revealing chromosome deletions in 3 and pathogenic mutations in 6 patients. Fourteen patients exhibited brain structure abnormalities such as intracranial hemorrhage and encephalomalacia. The birth history indicated various complications, including asphyxia/hypoxic-ischemic encephalopathy in 30 cases. Seizure manifestations included flexion of the limbs and nodding in 30 cases. Video EEG showed interictal hypsarrhythmia and an ictal voltage drop in 28 cases. The control group also consisted of 35 patients, with 18 boys and 17 girls, and a similar age of onset range (6 days to 1 year and 10 months) and average (4.57 ± 4.33 months). However, details regarding antiepileptic drugs, genetic testing, brain structure abnormalities, birth history, seizure manifestations, and video EEG were not provided for this group (Supplementary Table S1).

### Therapeutic effective rate of ACTH in the observation group

**Figure 1:**
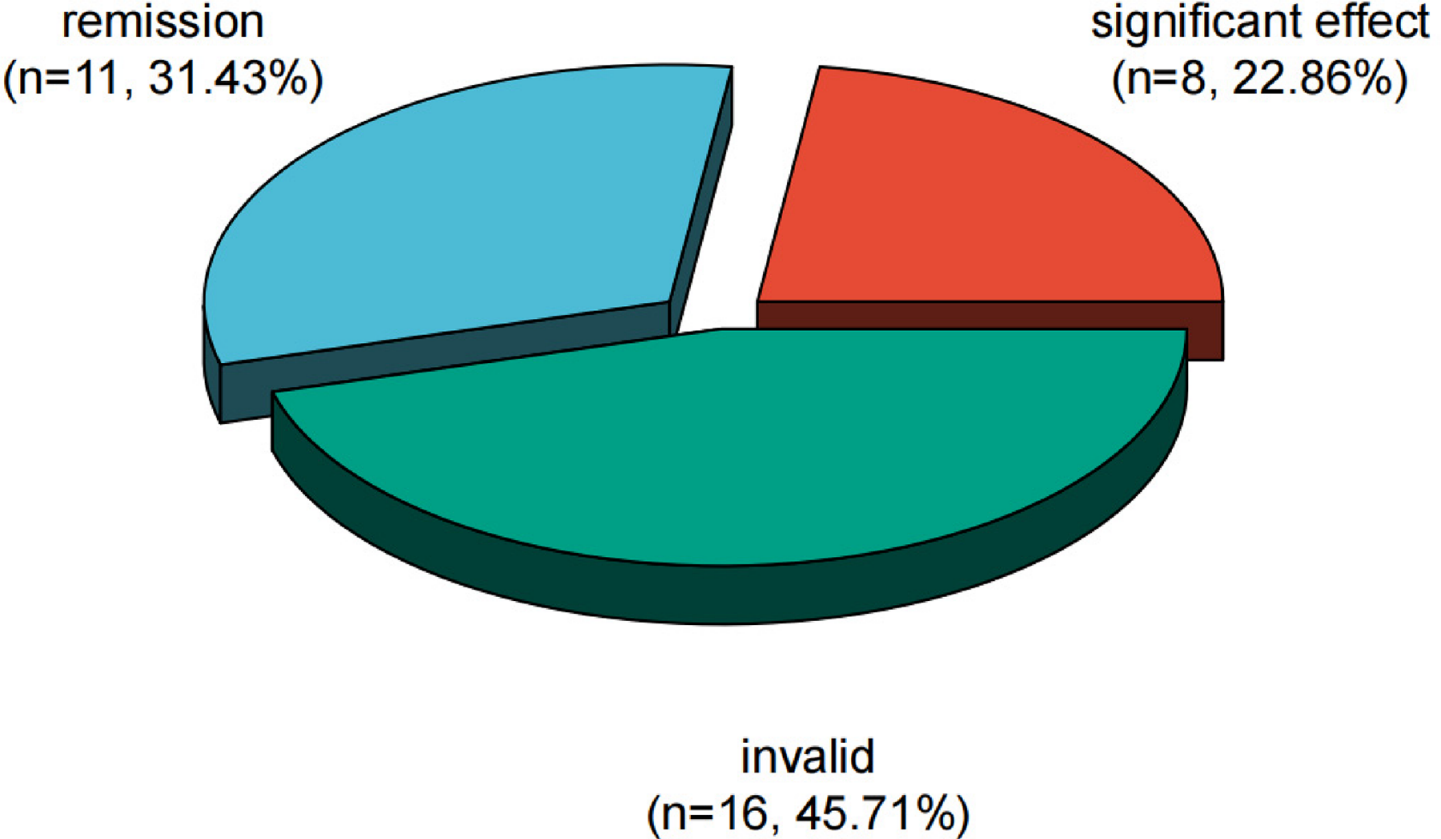
Therapeutic effects of ACTH in the observation group.

After therapy, the frequency of spasms in the observation group was counted and scored. The results showed that 8 cases were markedly effective, accounting for 22.86%; there were 11 cases of remission, accounting for 31.43%; 16 cases were invalid, accounting for 45.71%; and the total effective rate was 54.29% (Figure 1).

### Comparison of cytokine and immunoglobulin levels in peripheral blood between the two groups before therapy

Before treatment, we observed significant differences in the levels of immunoglobulins, lymphocyte subsets, and cytokines between the IS group and the control group. In the IS group, the serum levels of IgG and IgM were significantly higher (5.82 ± 1.55 and 0.89 ± 0.23 g/L, respectively), while the level of IgA was significantly lower (0.28 ± 0.29 g/L) compared to the control group (IgG: 4.73 ± 1.68 g/L, IgA: 0.42 ± 0.18 g/L, and IgM: 0.69 ± 0.29 g/L) (Table 1).

**Table 1:** Detection results of immunoglobulin and inflammatory cytokine levels between the two groups before treatment (mean and standard deviation $\bar{x}\;\;\pm \;s$, *n* = 35).

	Immunoglobulin level (g/L)			Inflammatory cytokine level		
	IgG	IgA	IgM	IL-1β (pg/L)	IL-2R (U/ml)	IL-6 (pg/ml)
IS group	5.82 ± 1.55	0.28 ± 0.29	0.89 ± 0.23	9.88 ± 5.82	1,425.97 ± 679.59	3.75 ± 2.57
Control group	4.73 ± 1.68	0.42 ± 0.18	0.69 ± 0.29	5.07 ± 0.30	510.89 ± 116.32	2.19 ± 0.70
*t*	2.81	2.31	3.17	4.88	7.85	3.47
*p*	**0.006**	**0.024**	**0.002**	**<0.001**	**<0.001**	**0.001**

Bold *p* values are statistically significant at *p* < 0.05.

In terms of lymphocyte subsets, the IS group had a significantly lower percentage of CD3^+^ T cells (56.13 ± 9.76%) and CD3^+^CD4^+^ T cells (35.46 ± 7.27%) but a higher percentage of CD3^−^CD19^+^ B cells (35.21 ± 11.18%) compared to the control group (CD3^+^ T cells: 67.60 ± 5.94%, CD3^+^CD4^+^ T cells: 43.35 ± 6.11%, and CD3^−^CD19^+^ B cells: 19.73 ± 6.69%). However, there were no significant differences in the percentages of CD3^+^CD8^+^ T cells and CD3^−^CD16^+^CD56^+^ NK cells and the ratio of CD3^+^CD4^+^/CD3^+^CD8^+^ between the two groups (Table 2).

**Table 2:** Results of lymphocyte count between two groups before treatment (mean and standard deviation $\bar{x}\;\;\pm \;s$, *n* = 35).

	Cell percentage					CD3^+^CD4^+^/CD3^+^CD8^+^
	CD3^+^	CD3^+^CD4^+^	CD3^+^CD8^+^	CD3^−^CD16^+^CD56^+^NK	CD3^−^CD19^+^	
IS group	56.13 ± 9.76	35.46 ± 7.27	18.78 ± 5.27	11.18 ± 6.90	35.21 ± 11.18	2.32 ± 0.75
Control group	67.60 ± 5.94	43.35 ± 6.11	20.60 ± 6.00	9.78 ± 3.99	19.73 ± 6.69	2.35 ± 1.02
*t*	5.94	4.91	1.35	1.04	7.03	0.15
*p*	**<0.001**	**<0.001**	0.183	0.301	**<0.001**	0.883

Bold *p* values are statistically significant at *p* < 0.05.

**Figure 2:**
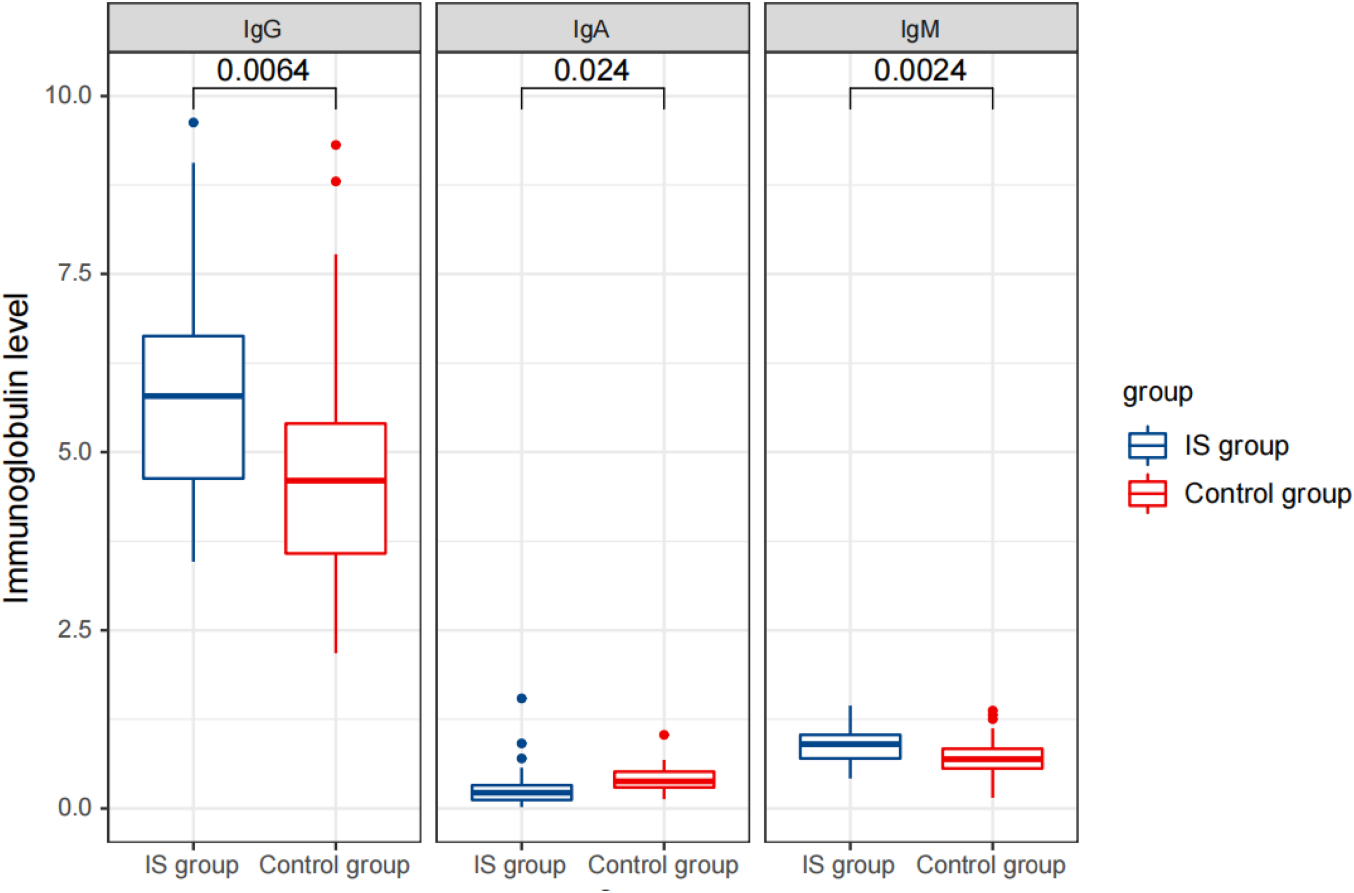
Comparison of detection results of immunoglobulin levels between the two groups before treatment.

**Figure 3:**
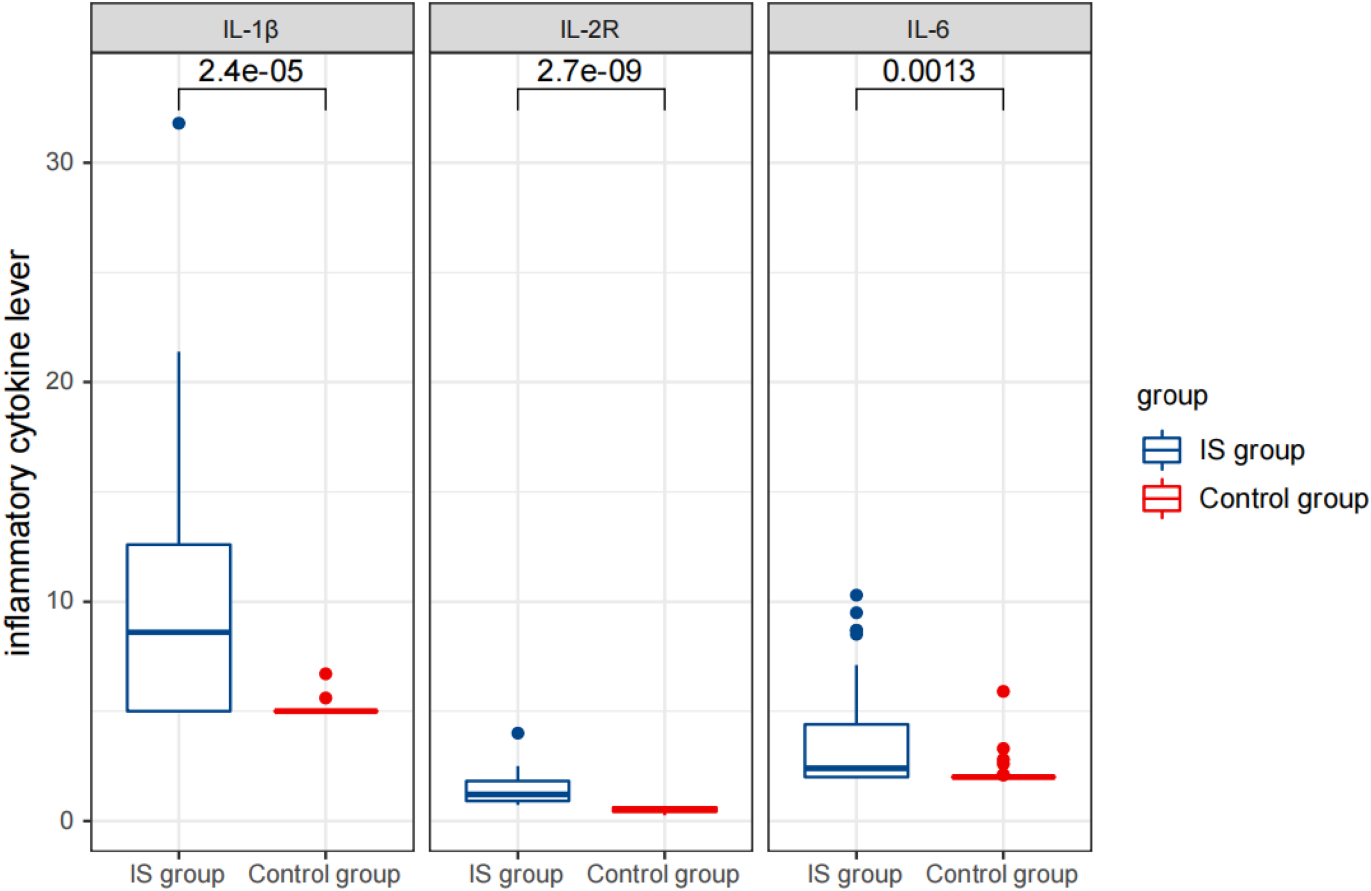
Comparison of detection results of inflammatory cytokine levels between the two groups before therapy.

**Figure 4:**
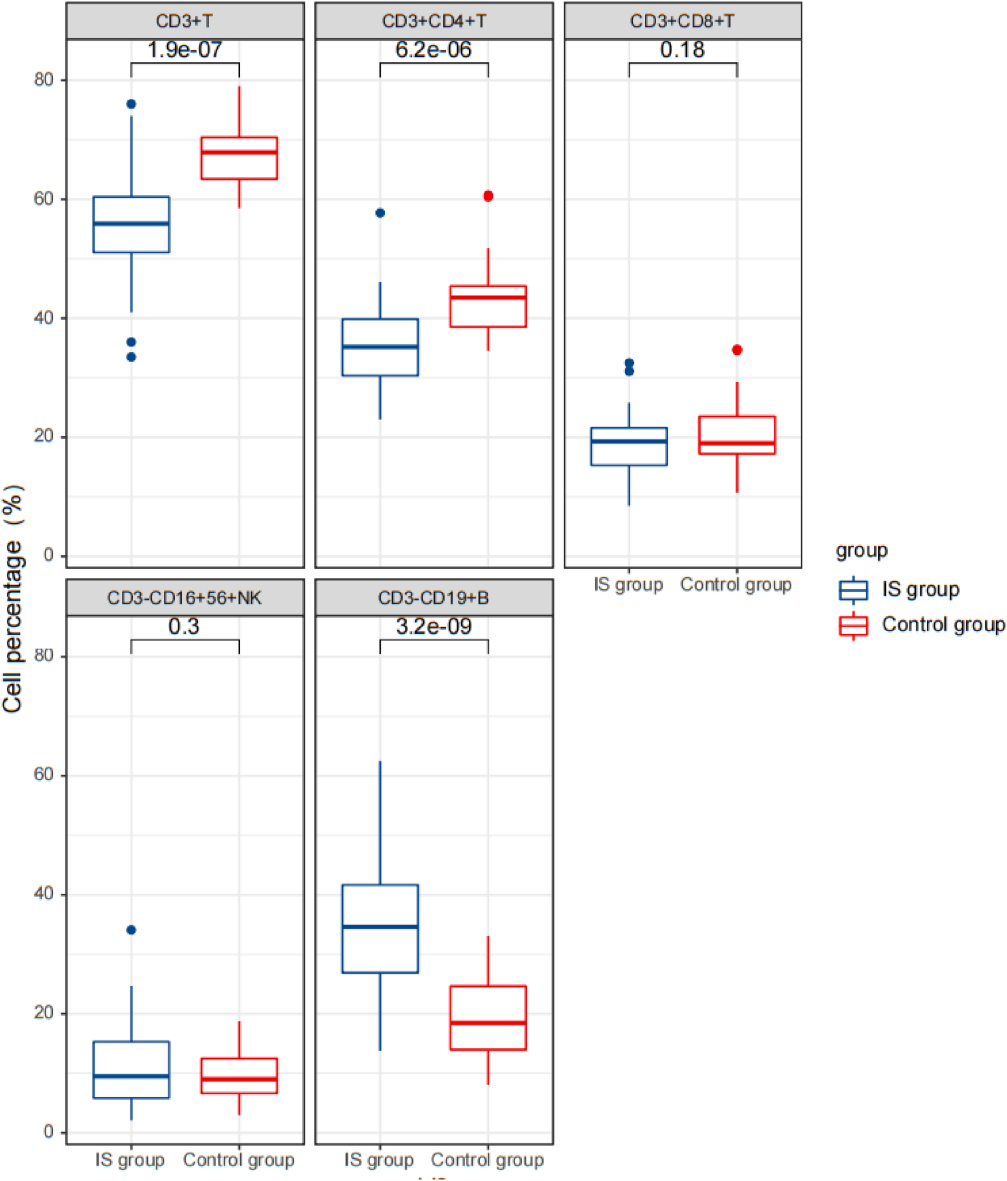
Comparison of lymphocyte count results between the two groups before therapy.

Furthermore, the IS group had significantly higher levels of cytokines IL-1 β (9.88 ± 5.82 pg/L), IL-2R (1,425.97 ± 679.59 U/ml), and IL-6 (3.75 ± 2.57 pg/ml) compared to the control group (IL-1 β: 5.07 ± 0.30 pg/L, IL-2R: 510.89 ± 116.32 U/ml, and IL-6: 2.19 ± 0.70 pg/ml) (Table 1) (Figures 2–4).

These findings suggest that children with IS have significant alterations in their immune profiles compared to healthy controls, which may contribute to the pathogenesis of the disease.

### Comparison of peripheral blood cytokine and immunoglobulin levels before and after treatment in the observation group

Before treatment, the serum levels of IgG, IgA, and IgM were 5.82 ± 1.55, 0.28 ± 0.29, and 0.89 ± 0.23 g/L, respectively. The proportions of CD3^+^ T cells, CD3^+^CD4^+^ T cells, CD3^+^CD8^+^ T cells, CD3^−^CD16^+^ CD56^+^ NK cells, and CD3^−^CD19^+^ B cells were 56.13 ± 9.76%, 35.46 ± 7.27%, 18.78 ± 5.27%, 11.18 ± 6.90%, and 19.73 ± 6.69%, respectively. The levels of cytokines IL-1 β, IL-2R, and IL-6 were 9.88 ± 5.82 pg/L, 1,425.97 ± 679.59 U/ml, and 3.75 ± 2.57 pg/ml, respectively.

After treatment, the serum levels of IgG, IgA, and IgM were 4.98 ± 1.67, 0.32 ± 0.32, and 0.85 ± 0.41 g/L, respectively. The proportions of CD3^+^ T cells, CD3^+^CD4^+^ T cells, CD3^+^CD8^+^ T cells, CD3^−^CD16^+^CD56^+^ NK cells, and CD3^−^CD19^+^ B cells were 62.24 ± 8.61%, 40.40 ± 7.27%, 18.52 ± 5.01%, 5.85 ± 2.80%, and 23.73 ± 7.88%, respectively. The levels of cytokines IL-1 β, IL-2R, and IL-6 were 6.65 ± 3.50 pg/L, 869.63 ± 476.79 U/ml, and 2.13 ± 0.21 pg/ml, respectively.

Statistical analysis revealed that after treatment, the serum IgG levels were significantly reduced (*p* < 0.05), while the IgA and IgM levels did not change significantly (*p* > 0.05). The percentages of CD3^+^ T cells and CD3^+^CD4^+^ T cells significantly increased, while the percentages of CD3^−^CD16^+^CD56^+^ NK cells and CD3^−^CD19^+^ B cells significantly decreased (*p* < 0.05). The percentages of CD3^+^CD8^+^ T cells and the ratio of CD3^+^CD4^+^/CD3^+^CD8^+^ did not change significantly (*p* > 0.05). The levels of cytokines IL-1 β, IL-2R, and IL-6 were significantly reduced (*p* < 0.05).

**Figure 5:**
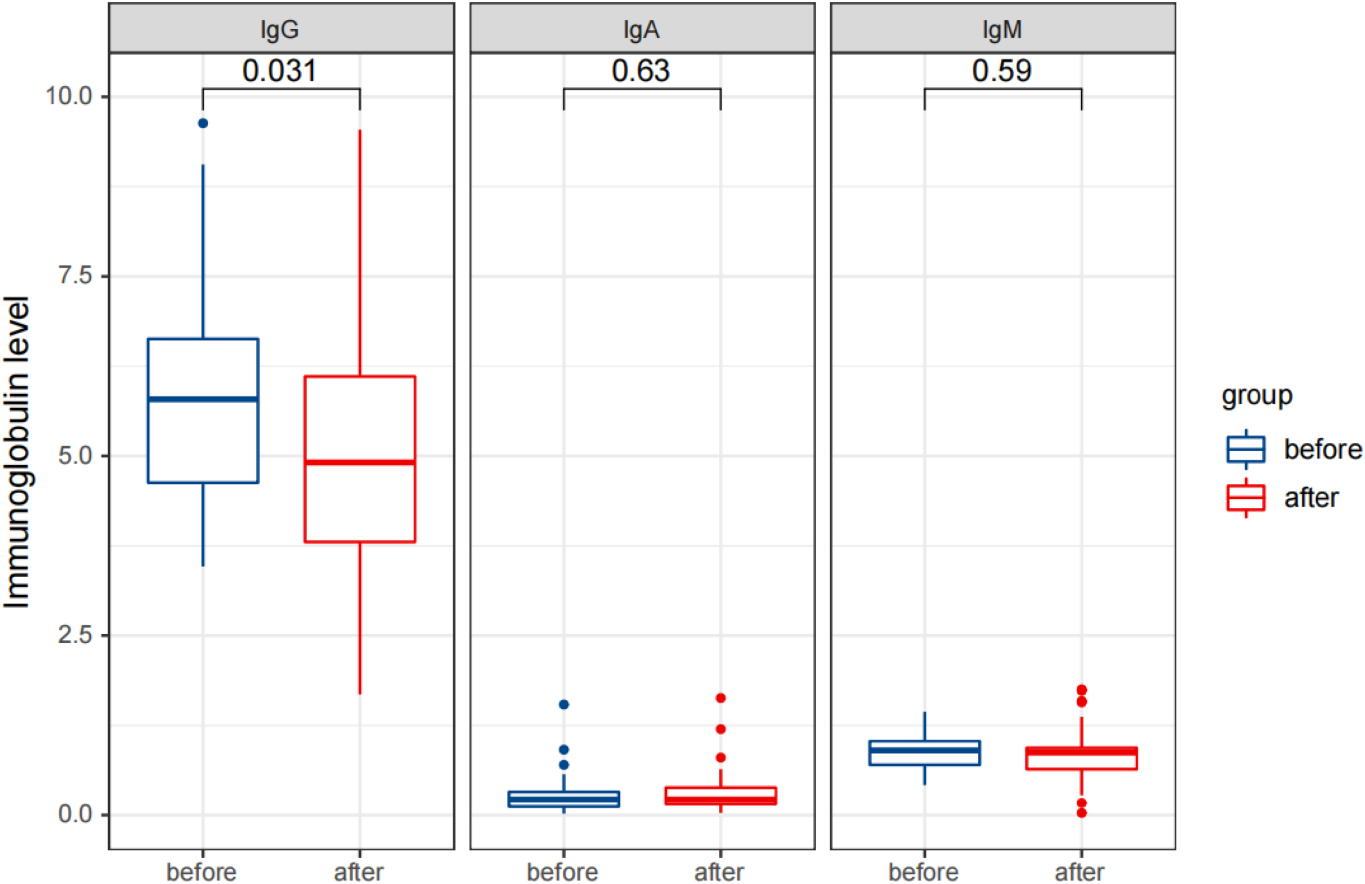
Comparison of detection results of immunoglobulin levels in the observation group before and after therapy.

**Figure 6:**
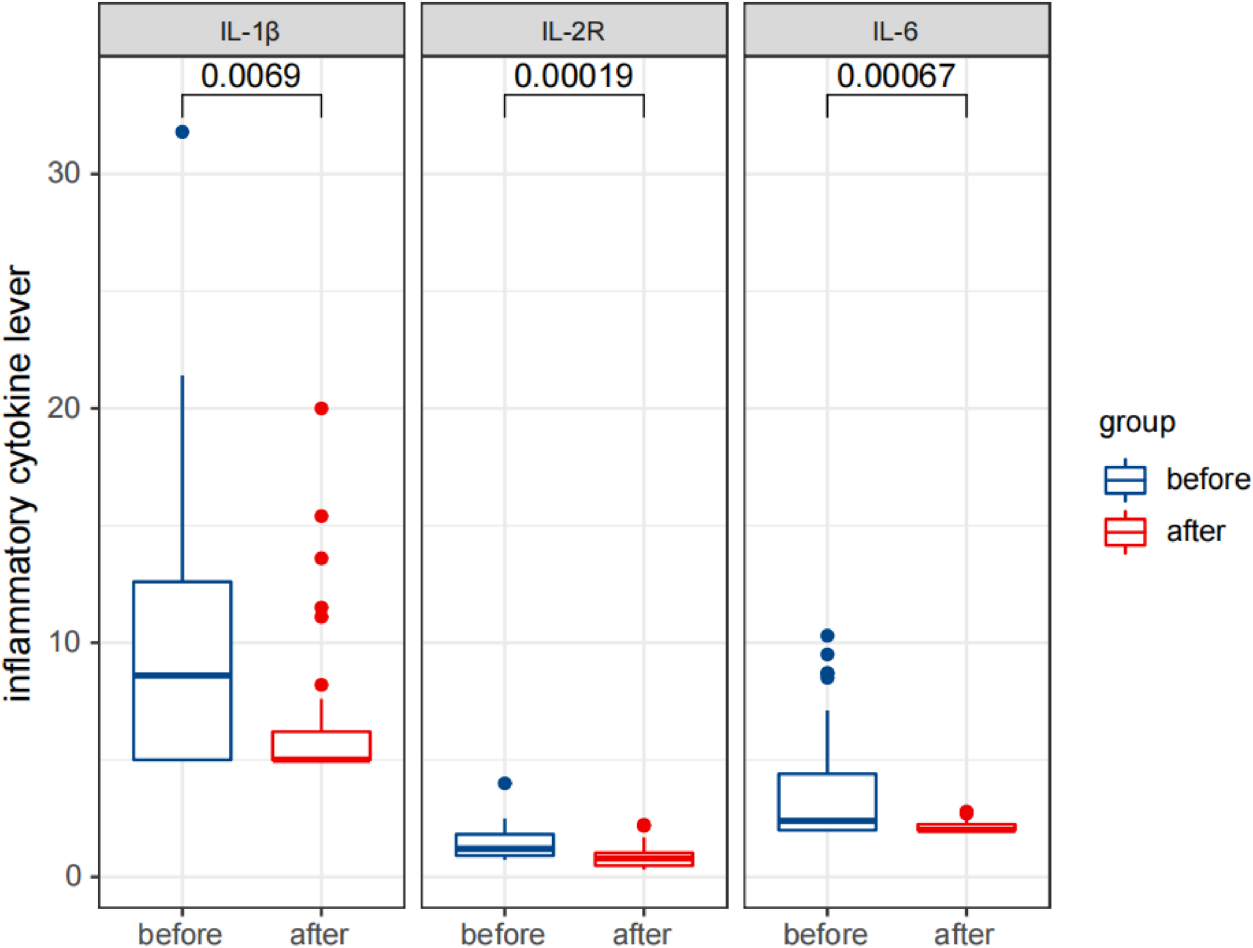
Comparison of detection results of inflammatory cytokine levels in the observation group before and after therapy.

**Figure 7:**
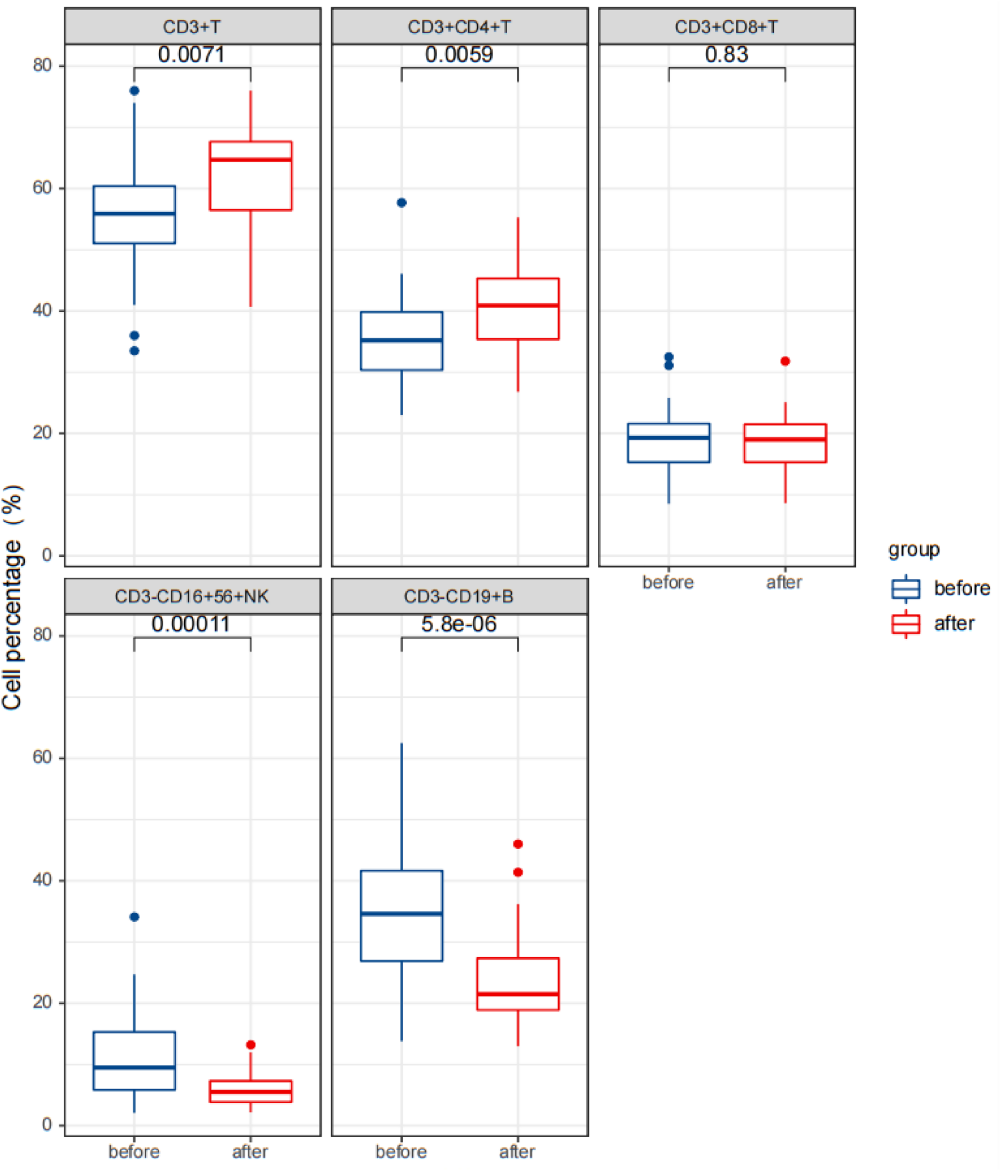
Comparison of results of lymphocyte count in the observation group before and after therapy.

These results are presented in Tables 3, 4, and Figures 5–7. Table 3 presents the detection results of immunoglobulin and inflammatory cytokine levels before and after treatment. Table 4 presents the results of the lymphocyte count before and after treatment. Figures 5–7 illustrate the comparison of immunoglobulin and inflammatory cytokine levels before and after treatment.

**Table 3:** Detection results of immunoglobulin and inflammatory cytokine levels in the IS group before and after treatment (mean and standard deviation $\bar{x}\;\;\pm \;s$, *n* = 35).

	Immunoglobulin level (g/L)			Inflammatory cytokine level		
	IgG	IgA	IgM	IL-1β (pg/L)	IL-2R (U/ml)	IL-6 (pg/ml)
Before	5.82 ± 1.55	0.28 ± 0.29	0.89 ± 0.23	9.88 ± 5.82	1,425.97 ± 679.59	3.75 ± 2.57
After	4.98 ± 1.67	0.32 ± 0.32	0.85 ± 0.41	6.65 ± 3.50	869.63 ± 476.79	2.13 ± 0.21
*t*	2.20	0.49	0.55	2.81	3.96	3.68
*p*	**0.031**	0.625	0.585	**0.007**	**<0.001**	**<0.001**

Bold *p* values are statistically significant at *p* < 0.05.

**Table 4:** Results of lymphocyte count in the IS group before and after treatment (mean and standard deviation $\bar{x}\;\;\pm \;s$, *n* = 35).

	Cell percentage					CD3^+^CD4^+^/CD3^+^CD8^+^
	CD3^+^	CD3^+^CD4^+^	CD3^+^CD8^+^	CD3^−^CD16^+^CD56^+^NK	CD3^−^CD19^+^	
Before	56.13 ± 9.76	35.46 ± 7.27	18.78 ± 5.27	11.18 ± 6.90	35.21 ± 11.18	2.32 ± 0.75
After	62.24 ± 8.61	40.40 ± 7.27	18.52 ± 5.01	5.85 ± 2.80	23.73 ± 7.88	2.05 ± 0.72
*t*	2.78	2.84	0.21	4.23	4.96	1.49
*p*	**0.007**	**0.006**	0.833	**<0.001**	**<0.001**	0.142

Bold *p* values are statistically significant at *p* < 0.05.

## Discussion

IS, also known as West's syndrome (WS) (11), is a severe infantile epileptic spasms syndrome (IESS) that is clinically characterized by spasticity, neurodevelopmental degeneration, and high-grade dysrhythmia on the EEG (12). According to statistics, 1 in every 2,000–4,000 infants suffers from this disease (13). The disease usually attacks within the first 2 years after birth (14), with a peak onset at 4–7 months, and about a quarter of patients will spontaneously stop spasticity within 1 year after onset (15). The treatment of IS is difficult and has a high incidence rate. At present, the most commonly used clinical therapeutic drugs include ACTH, corticosteroids, and vigabatrin (VGB) (16, 17). In the United States, the preferred drug for IS has been ACTH, which is generally considered to be more effective than corticosteroids. Adrenocorticotropic hormone can not only change the long-term prognosis of cryptogenic IS but also help improve the clinical symptoms of children with IS (18).

Because early diagnosis and timely therapy are significant for the rehabilitation and prognosis of children with IS, further research on the methods of IS diagnosis and evaluation of drug efficacy has become an urgent problem to be solved. Therefore, ACTH was selected as the clinical treatment drug in this study, and the related factor levels in the venous serum of children with IS, healthy children, and children with IS before and after therapy were compared and analyzed.

The immune deficiency in children with IS is mainly manifested by sensitization to 2,4-dinitrochlorobenzene (DNCB), intradermal reaction to Phytohemagglutinin (PHA), inhibition of leukocyte migration, blast transformation of lymphocytes (low CD4^+^/CD8^+^ ratio), T lymphocytes (low CD3^+^, low CD4^+^, and high CD8^+^), and B lymphocytes (high CD3^−^CD19^+^) in peripheral blood, and changes in the levels of serum immunoglobulins (high levels of IgG and IgM and low levels of IgA) (18, 19).

Currently, many research studies have shown that the occurrence and development of IS are related to immunity (20). The serum levels of immunoglobulins (IgG, IgA, and IgM) are an intuitive factor reflecting the immune function of the body. Immunoglobulins that enter the brain through the blood–brain barrier can inhibit the expression of cytokines such as C-Fos and r-interferon, which are closely related to IS, through a wide range of immunomodulatory effects and improve the activity of natural killer cells (21). This study found a significant distinction in the immunoglobulin levels in the serum of children with IS compared to healthy children, manifested by increased levels of IgG and IgM and decreased levels of IgA (22). The detection results of immunoglobulin levels in this study revealed that compared to the healthy children (control one), the levels of immunoglobulins IgG and IgM in the serum of children with IS (observation one) were significantly increased, while IgA levels were significantly reduced, with statistically significant differences (*p* < 0.05), which was consistent with the literature research results. After ACTH therapy, the level of immunoglobulin IgG in the serum of children with IS was significantly reduced (*p* < 0.05), but the IgA and IgM levels did not change significantly (*p *> 0.05). Therefore, it is presumed that the therapeutic mechanism of ACTH on IS does not involve changes in immunoglobulin levels.

Peripheral blood T lymphocyte subsets can reflect human cellular immune function. CD3^+^ T lymphocytes are a common marker on the surface of all T cells, and their content represents the levels of total T lymphocytes and reflects the functional state of cellular immunity (23); CD4^+^ T cells, known as helper T lymphocytes, are the most important hub cells to regulate the immune response. They release a variety of cytokines to participate in the immune response, and a decline in their proportion indicates inhibited T-cell immune function (24). CD8^+^ T cells are suppressive T lymphocytes that play a negative regulatory role in the immune response by inhibiting the function of CD4^+^ cells (23, 25). At the same time, studies have found that children with IS also have immune dysfunction of B lymphocytes (25). B lymphocytes secrete regulatory cytokines and present antigens to T cells. CD19^+^ B lymphocytes, a member of the immunoglobulin superfamily, play a significant role in the proliferation, differentiation, and activation of B lymphocytes (26). NK cells serve as the first line of defense in the body and can play an important role in immunity. CD56, a neural cell adhesion molecule, is mainly expressed on the surface of NK cells (CD3^−^CD56^+^) and innate immune-like T cells (CD3^+^CD56^+^), including Natural Killer T cells (NKT) cells in the peripheral blood (27); increased levels of CD3^−^CD56^+^ NK cells can be detected in immunodeficient children. Previous studies have shown that the proportion of CD4^+^ T cells in the peripheral blood of children with IS was significantly reduced, the proportion of CD8^+^ T cells was significantly increased, the ratio of CD4^+^/CD8^+^ was reduced (28), and the proportion of CD19^+^ B lymphocytes was significantly increased (25). The results of this study revealed that compared to healthy children (control group), the percentage of CD3^−^CD19^+^ B cells in the serum of children with IS (observation group) was significantly increased, and the percentages of CD3^+^ T cells and CD3^+^CD4^+^ T cells were significantly reduced (*p* < 0.05). After ACTH treatment, the percentages of CD3^+^ T cells and CD3^+^CD4^+^ T cells in the serum of children with IS were significantly increased, while the percentages of CD3^−^CD16^+^CD56^+^ NK cells and CD3^−^CD19^+^ B cells were significantly decreased (*p* < 0.05), which was consistent with the results of previous literature research.

The data indicate that inflammatory processes are involved in the occurrence and development of IS through many mechanisms, with proinflammatory cytokines (PICs) playing a key role in brain inflammatory responses. Evidence suggests that the onset of IS is associated with increased PIC levels, including IL-1 β, IL-6, TNF-α, and other cytokines (29, 30). In this study, the IL-2R, IL-6, and IL-1 β levels were measured, and the results showed that compared with healthy kids (control group), the levels of cytokines IL-1β, IL-2R, and IL-6 in the serum of children with IS (observation group) were significantly increased (*p* < 0.05). After ACTH therapy, the levels of IL-1β, IL-2R, and IL-6 in the serum of children with IS were significantly decreased (*p* < 0.05), and the above results were consistent with the literature.

While our study provides valuable insights into the effects of ACTH therapy on peripheral blood cytokine and immunoglobulin levels, several limitations should be acknowledged. First, our study was conducted with a relatively small sample size, which may limit the generalizability of our findings. Future studies with larger sample sizes are needed to confirm our results. Second, our study was observational. Therefore, we cannot establish causality between ACTH therapy and changes in cytokine and immunoglobulin levels. Randomized controlled trials are needed to establish a causal relationship. Third, we only measured cytokine and immunoglobulin levels at two time points—before and after treatment. This does not allow us to track the dynamic changes in these levels over the course of treatment. Future studies could consider more frequent measurements to capture these dynamics. Finally, we did not control for potential confounding factors such as age, sex, and comorbid conditions, which could influence cytokine and immunoglobulin levels. Future research should consider these factors in their analyses. Despite these limitations, we believe our study contributes to the literature and can serve as a basis for future research in this area.

In this study, we observed significant changes in the levels of peripheral blood cytokines and immunoglobulins following ACTH therapy. These findings suggest a potential role for immune dysregulation in the pathogenesis of IS. Our study adds to this body of knowledge by providing quantitative data on these changes and their response to ACTH therapy. The observed reduction in serum IgG levels and the increase in CD3+ T cells and CD3+ CD4+ T cells after treatment suggest that ACTH therapy may modulate the immune response in children with IS. Our findings have important implications for clinical practice. The significant changes in immune markers following ACTH therapy suggest that these may serve as therapeutic targets or biomarkers of treatment response. However, further research is needed to validate these findings in larger cohorts and to explore the underlying mechanisms.

In conclusion, this study believes that the abnormal changes in serum levels of immunoglobulins (IgG, IgA, and IgM), lymphocytes (CD3^+^ T, CD3^+^CD4^+^ T, and CD3^−^CD19^+^ B), and inflammatory factors (IL-1 β, IL-2R, and IL-6) can be used as auxiliary diagnostic indicators of IS by detecting the levels of lymphocytes, immunoglobulins, and cytokines in healthy children and children with IS before and after ACTH treatment; also, lymphocytes (CD3^+^ T, CD3^+^CD4^+^ T, CD3^−^CD16^+^CD56^+^ NK, and CD3^−^CD19^+^ B) and inflammatory factor (IL-1 β, IL-2R, and IL-6) levels can be used as detection indicators to evaluate the therapeutic effects of ACTH. However, the immunological pathogenesis of IS and the influence of immune factors on the occurrence, development, and treatment of IS have not been clarified. Therefore, it is necessary to further study and explore the immunological mechanism of IS and further understand the etiology and development process of IS and the treatment mechanism of ACTH to provide new methods and thoughts for the diagnosis, therapy, and prognosis of IS.

## Data Availability

The original contributions presented in the study are included in the article/**Supplementary Material**, further inquiries can be directed to the corresponding author.
