# Supplementary material for: Application of peripheral blood cytokine and immunoglobulin detection in ACTH therapy for the treatment of infantile spasms
Source: Front Pediatr. 2024 Jul 10;12:1365917. doi: 10.3389/fped.2024.1365917 (PMC11270505; doi:10.3389/fped.2024.1365917)
Supplement: Supplementary file 1 [file Table1.docx]

IS Group comprised 35 patients, including 20 males and 15 females, with an age of onset ranging from 6 days to 1 year and 10 months (average 4.98 ± 4.19 months). Eighteen patients had previously taken 1-3 antiepileptic drugs without successful seizure control. Genetic testing was conducted on 16 patients, revealing chromosome deletions in 3 and pathogenic mutations in 6. Fourteen patients exhibited brain structure abnormalities such as intracranial hemorrhage and encephalomalacia. Birth history indicated various complications, including asphyxia/hypoxic-ischemic encephalopathy in 30 cases. Seizure manifestations included flexion of limbs and nodding in 30 cases. Video EEG showed interictal hypsarrhythmia and ictal voltage drop in 28 cases.The Control Group also consisted of 35 patients, with 18 males and 17 females, and a similar age of onset range and average (6 days to 1 year and 10 months; 4.57 ± 4.33 months). However, details regarding antiepileptic drugs, genetic testing, brain structure abnormalities, birth history, seizure manifestations, and video EEG were not provided for this group.

Supplementary Table1 The demographic and clinical characteristics of two Group

| Characteristics | Details | |
| --- | --- | --- |
|  | IS Group | Control Group |
| Total Number of Patients  Age of Onset  Antiepileptic Drugs  Genetic Testing  Brain Structure Abnormalities  Birth History  Seizure Manifestations  Video EEG | 35 (20 males, 15 females)  6 days to 1 year 10 months  (4.98 ± 4.19 months)  18 patients had taken 1-3 antiepileptic drugs before ACTH treatment, seizures were uncontrolled  16 patients underwent genetic testing, 3 had chromosome deletions, 6 had pathogenic mutations  14 patients (intracranial hemorrhage, encephalomalacia, supratentorial ventricular enlargement, widened extracerebral space)  30 cases with asphyxia/hypoxic-ischemic encephalopathy, 10 premature, 2 multiple births, 3 intracranial hemorrhage, 5 intracranial infections, 18 unknown etiology  Flexion of limbs, nodding: 30 cases, Extension of limbs, head tilting back: 20 cases, Asymmetric spasms: 8 cases  Interictal hypsarrhythmia and ictal voltage drop: 28 cases, Interictal paroxysmal hypsarrhythmia: 3 cases | 35 (18 males, 17 females)  6 days to 1 year 10 months  (4.57 ± 4.33 months)  -  -  -  -  -  - |
